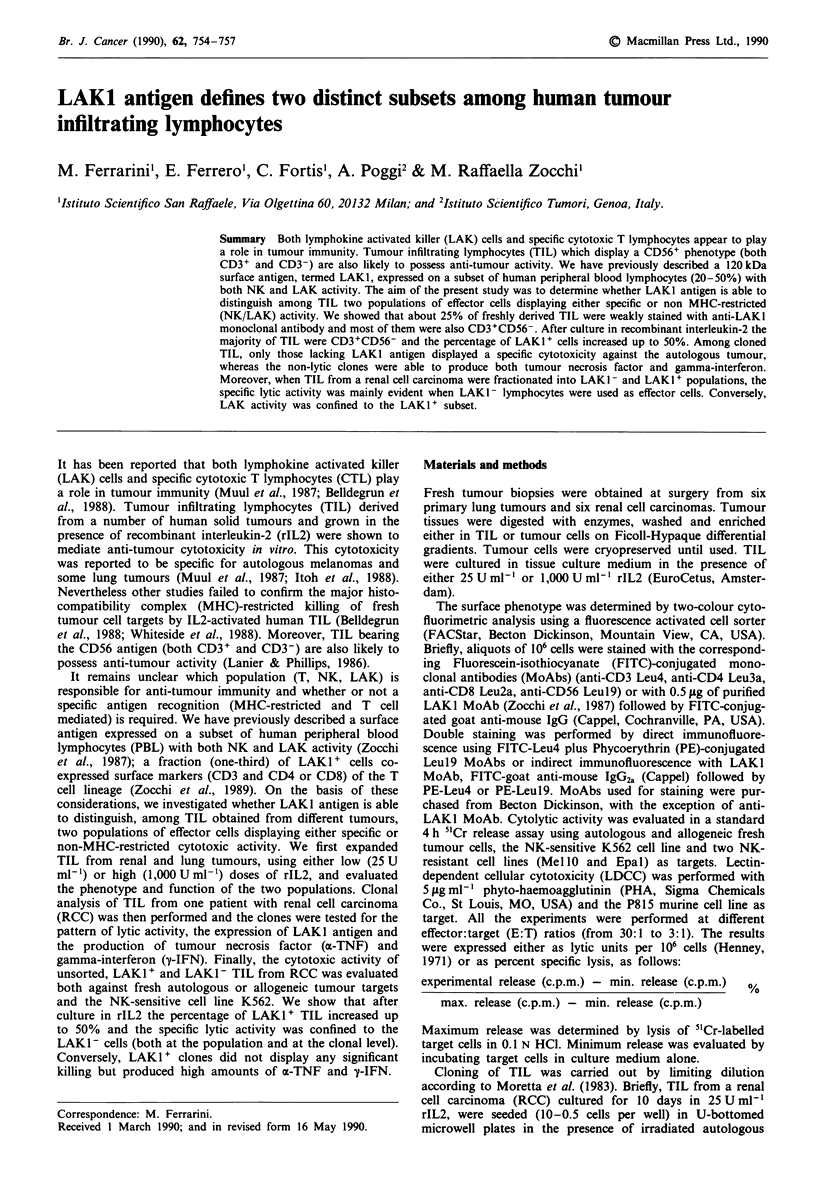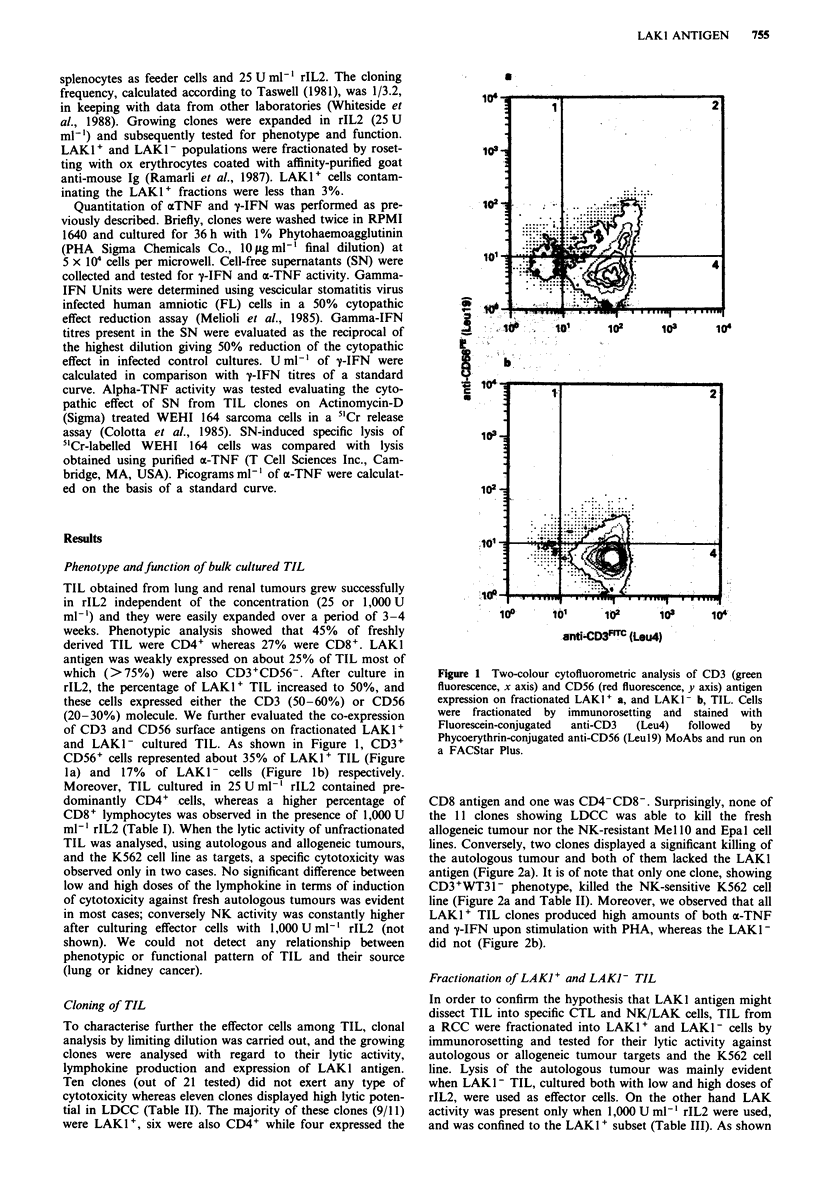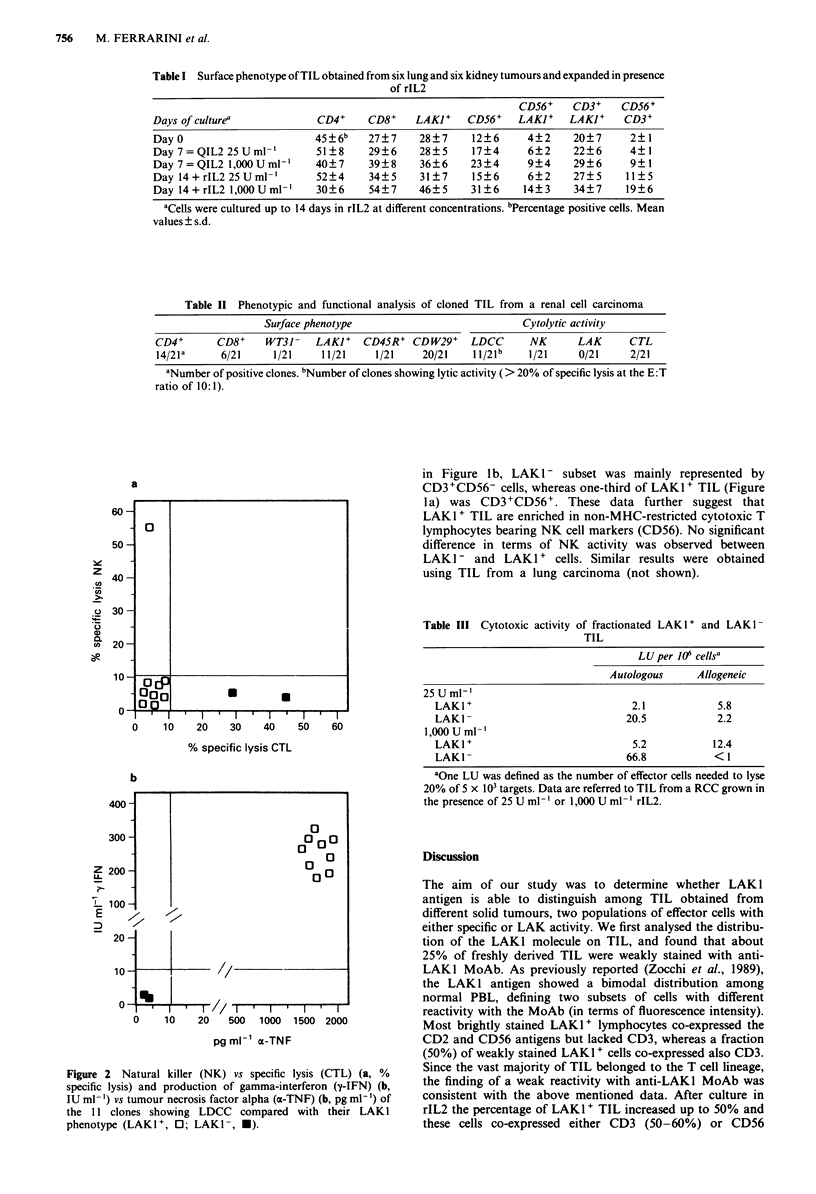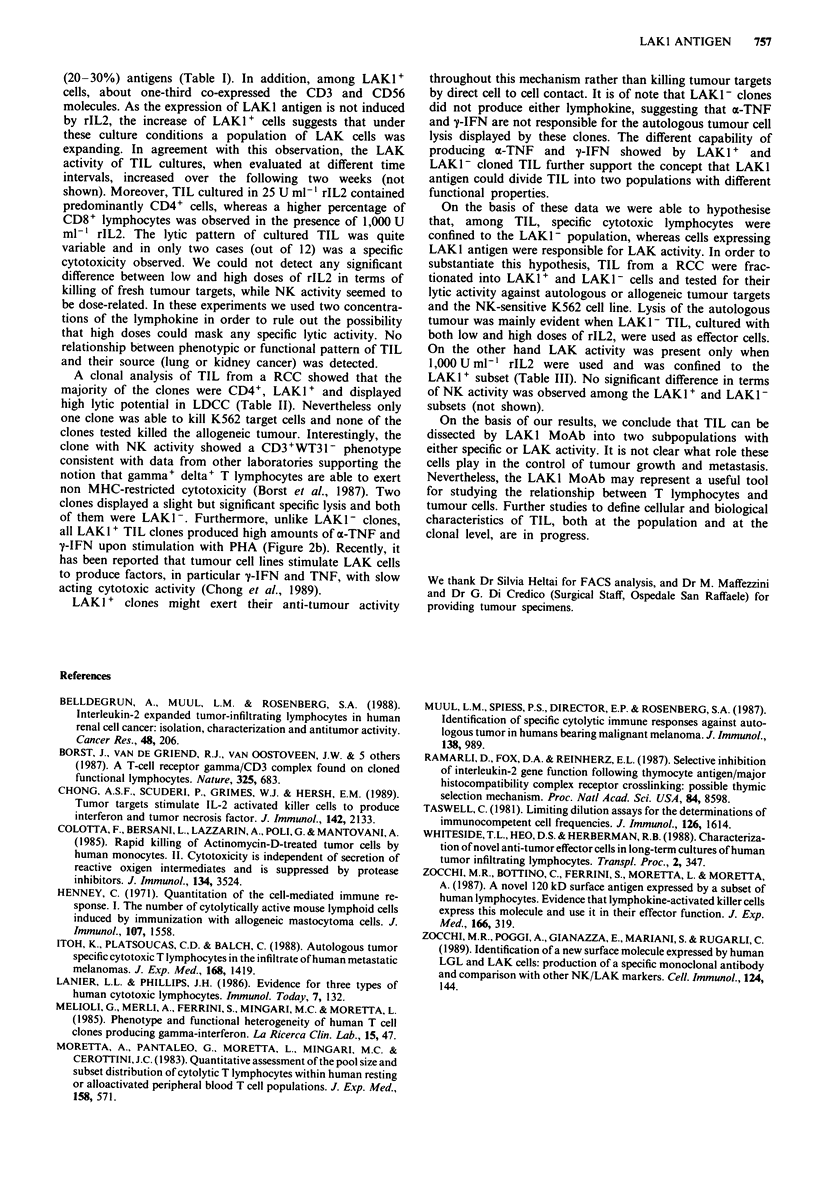# LAK1 antigen defines two distinct subsets among human tumour infiltrating lymphocytes.

**DOI:** 10.1038/bjc.1990.372

**Published:** 1990-11

**Authors:** M. Ferrarini, E. Ferrero, C. Fortis, A. Poggi, M. R. Zocchi

**Affiliations:** Istituto Scientifico San Raffaele, Milan, Italy.

## Abstract

Both lymphokine activated killer (LAK) cells and specific cytotoxic T lymphocytes appear to play a role in tumour immunity. Tumour infiltrating lymphocytes (TIL) which display a CD56+ phenotype (both CD3+ and CD3-) are also likely to possess anti-tumour activity. We have previously described a 120 kDa surface antigen, termed LAK1, expressed on a subset of human peripheral blood lymphocytes (20-50%) with both NK and LAK activity. The aim of the present study was to determine whether LAK1 antigen is able to distinguish among TIL two populations of effector cells displaying either specific or non MHC-restricted (NK/LAK) activity. We showed that about 25% of freshly derived TIL were weakly stained with anti-LAK1 monoclonal antibody and most of them were also CD3+ CD56-. After culture in recombinant interleukin-2 the majority of TIL were CD3+ CD56- and the percentage of LAK1+ cells increased up to 50%. Among cloned TIL, only those lacking LAK1 antigen displayed a specific cytotoxicity against the autologous tumour, whereas the non-lytic clones were able to produce both tumour necrosis factor and gamma-interferon. Moreover, when TIL from a renal cell carcinoma were fractionated into LAK1- and LAK1+ populations, the specific lytic activity was mainly evident when LAK1- lymphocytes were used as effector cells. Conversely, LAK activity was confined to the LAK1+ subset.


Br. J. Cancer (1990), 62, 754-757 © Macmillan Press Ltd., 1990

### LAK1 antigen defines two distinct subsets among human tumour infiltrating lymphocytes

M. Ferrarini[1], E. Ferrero[1], C. Fortis[1], A. Poggi[2] & M. Raffaella Zocchi[1]

[1]*Istituto Scientifico San Raffaele, Via Olgettina 60, 20132 Milan; and* [2]*Istituto Scientifico Tumori, Genoa, Italy.*

**Summary** Both lymphokine activated killer (LAK) cells and specific cytotoxic T lymphocytes appear to play a role in tumour immunity. Tumour infiltrating lymphocytes (TIL) which display a $CD56^+$ phenotype (both $CD3^+$ and $CD3^-$) are also likely to possess anti-tumour activity. We have previously described a 120 kDa surface antigen, termed LAK1, expressed on a subset of human peripheral blood lymphocytes (20-50%) with both NK and LAK activity. The aim of the present study was to determine whether LAK1 antigen is able to distinguish among TIL two populations of effector cells displaying either specific or non MHC-restricted (NK/LAK) activity. We showed that about 25% of freshly derived TIL were weakly stained with anti-LAK1 monoclonal antibody and most of them were also $CD3^+CD56^-$. After culture in recombinant interleukin-2 the majority of TIL were $CD3^+CD56^-$ and the percentage of $LAK1^+$ cells increased up to 50%. Among cloned TIL, only those lacking LAK1 antigen displayed a specific cytotoxicity against the autologous tumour, whereas the non-lytic clones were able to produce both tumour necrosis factor and gamma-interferon. Moreover, when TIL from a renal cell carcinoma were fractionated into $LAK1^-$ and $LAK1^+$ populations, the specific lytic activity was mainly evident when $LAK1^-$ lymphocytes were used as effector cells. Conversely, LAK activity was confined to the $LAK1^+$ subset.

It has been reported that both lymphokine activated killer (LAK) cells and specific cytotoxic T lymphocytes (CTL) play a role in tumour immunity (Muul *et al.*, 1987; Belldegrun *et al.*, 1988). Tumour infiltrating lymphocytes (TIL) derived from a number of human solid tumours and grown in the presence of recombinant interleukin-2 (rIL2) were shown to mediate anti-tumour cytotoxicity *in vitro*. This cytotoxicity was reported to be specific for autologous melanomas and some lung tumours (Muul *et al.*, 1987; Itoh *et al.*, 1988). Nevertheless other studies failed to confirm the major histocompatibility complex (MHC)-restricted killing of fresh tumour cell targets by IL2-activated human TIL (Belldegrun *et al.*, 1988; Whiteside *et al.*, 1988). Moreover, TIL bearing the CD56 antigen (both $CD3^+$ and $CD3^-$) are also likely to possess anti-tumour activity (Lanier & Phillips, 1986).

It remains unclear which population (T, NK, LAK) is responsible for anti-tumour immunity and whether or not a specific antigen recognition (MHC-restricted and T cell mediated) is required. We have previously described a surface antigen expressed on a subset of human peripheral blood lymphocytes (PBL) with both NK and LAK activity (Zocchi *et al.*, 1987); a fraction (one-third) of $LAK1^+$ cells co-expressed surface markers (CD3 and CD4 or CD8) of the T cell lineage (Zocchi *et al.*, 1989). On the basis of these considerations, we investigated whether LAK1 antigen is able to distinguish, among TIL obtained from different tumours, two populations of effector cells displaying either specific or non-MHC-restricted cytotoxic activity. We first expanded TIL from renal and lung tumours, using either low (25 U $ml^{-1}$) or high (1,000 U $ml^{-1}$) doses of rIL2, and evaluated the phenotype and function of the two populations. Clonal analysis of TIL from one patient with renal cell carcinoma (RCC) was then performed and the clones were tested for the pattern of lytic activity, the expression of LAK1 antigen and the production of tumour necrosis factor (α-TNF) and gamma-interferon (γ-IFN). Finally, the cytotoxic activity of unsorted, $LAK1^+$ and $LAK1^-$ TIL from RCC was evaluated both against fresh autologous or allogeneic tumour targets and the NK-sensitive cell line K562. We show that after culture in rIL2 the percentage of $LAK1^+$ TIL increased up to 50% and the specific lytic activity was confined to the $LAK1^-$ cells (both at the population and at the clonal level). Conversely, $LAK1^+$ clones did not display any significant killing but produced high amounts of α-TNF and γ-IFN.

Correspondence: M. Ferrarini.
Received 1 March 1990; and in revised form 16 May 1990.

### Materials and methods

Fresh tumour biopsies were obtained at surgery from six primary lung tumours and six renal cell carcinomas. Tumour tissues were digested with enzymes, washed and enriched either in TIL or tumour cells on Ficoll-Hypaque differential gradients. Tumour cells were cryopreserved until used. TIL were cultured in tissue culture medium in the presence of either 25 U $ml^{-1}$ or 1,000 U $ml^{-1}$ rIL2 (EuroCetus, Amsterdam).

The surface phenotype was determined by two-colour cytofluorimetric analysis using a fluorescence activated cell sorter (FACStar, Becton Dickinson, Mountain View, CA, USA). Briefly, aliquots of $10^6$ cells were stained with the corresponding Fluorescein-isothiocyanate (FITC)-conjugated monoclonal antibodies (MoAbs) (anti-CD3 Leu4, anti-CD4 Leu3a, anti-CD8 Leu2a, anti-CD56 Leu19) or with 0.5 μg of purified LAK1 MoAb (Zocchi *et al.*, 1987) followed by FITC-conjugated goat anti-mouse IgG (Cappel, Cochranville, PA, USA). Double staining was performed by direct immunofluorescence using FITC-Leu4 plus Phycoerythrin (PE)-conjugated Leu19 MoAbs or indirect immunofluorescence with LAK1 MoAb, FITC-goat anti-mouse $IgG_{2a}$ (Cappel) followed by PE-Leu4 or PE-Leu19. MoAbs used for staining were purchased from Becton Dickinson, with the exception of anti-LAK1 MoAb. Cytolytic activity was evaluated in a standard 4 h $^{51}Cr$ release assay using autologous and allogeneic fresh tumour cells, the NK-sensitive K562 cell line and two NK-resistant cell lines (Mel10 and Epa1) as targets. Lectin-dependent cellular cytotoxicity (LDCC) was performed with 5 μg $ml^{-1}$ phyto-haemoagglutinin (PHA, Sigma Chemicals Co., St Louis, MO, USA) and the P815 murine cell line as target. All the experiments were performed at different effector:target (E:T) ratios (from 30:1 to 3:1). The results were expressed either as lytic units per $10^6$ cells (Henney, 1971) or as percent specific lysis, as follows:

$$\frac{\text{experimental release (c.p.m.)} - \text{min. release (c.p.m.)}}{\text{max. release (c.p.m.)} - \text{min. release (c.p.m.)}} \%$$

Maximum release was determined by lysis of $^{51}Cr$-labelled target cells in 0.1 N HCl. Minimum release was evaluated by incubating target cells in culture medium alone.

Cloning of TIL was carried out by limiting dilution according to Moretta *et al.* (1983). Briefly, TIL from a renal cell carcinoma (RCC) cultured for 10 days in 25 U $ml^{-1}$ rIL2, were seeded (10-0.5 cells per well) in U-bottomed microwell plates in the presence of irradiated autologous splenocytes as feeder cells and 25 U $ml^{-1}$ rIL2. The cloning frequency, calculated according to Taswell (1981), was 1/3.2, in keeping with data from other laboratories (Whiteside *et al.*, 1988). Growing clones were expanded in rIL2 (25 U $ml^{-1}$) and subsequently tested for phenotype and function. $LAK1^+$ and $LAK1^-$ populations were fractionated by rosetting with ox erythrocytes coated with affinity-purified goat anti-mouse Ig (Ramarli *et al.*, 1987). $LAK1^+$ cells contaminating the $LAK1^+$ fractions were less than 3%.

Quantitation of αTNF and γ-IFN was performed as previously described. Briefly, clones were washed twice in RPMI 1640 and cultured for 36 h with 1% Phytohaemoagglutinin (PHA Sigma Chemicals Co., 10 μg $ml^{-1}$ final dilution) at $5 \times 10^4$ cells per microwell. Cell-free supernatants (SN) were collected and tested for γ-IFN and α-TNF activity. Gamma-IFN Units were determined using vescicular stomatitis virus infected human amniotic (FL) cells in a 50% cytopathic effect reduction assay (Melioli *et al.*, 1985). Gamma-IFN titres present in the SN were evaluated as the reciprocal of the highest dilution giving 50% reduction of the cytopathic effect in infected control cultures. U $ml^{-1}$ of γ-IFN were calculated in comparison with γ-IFN titres of a standard curve. Alpha-TNF activity was tested evaluating the cytopathic effect of SN from TIL clones on Actinomycin-D (Sigma) treated WEHI 164 sarcoma cells in a $^{51}Cr$ release assay (Colotta *et al.*, 1985). SN-induced specific lysis of $^{51}Cr$-labelled WEHI 164 cells was compared with lysis obtained using purified α-TNF (T Cell Sciences Inc., Cambridge, MA, USA). Picograms $ml^{-1}$ of α-TNF were calculated on the basis of a standard curve.

### Results

### *Phenotype and function of bulk cultured TIL*

TIL obtained from lung and renal tumours grew successfully in rIL2 independent of the concentration (25 or 1,000 U $ml^{-1}$) and they were easily expanded over a period of 3–4 weeks. Phenotypic analysis showed that 45% of freshly derived TIL were $CD4^+$ whereas 27% were $CD8^+$. LAK1 antigen was weakly expressed on about 25% of TIL most of which (>75%) were also $CD3^+CD56^-$. After culture in rIL2, the percentage of $LAK1^+$ TIL increased to 50%, and these cells expressed either the CD3 (50–60%) or CD56 (20–30%) molecule. We further evaluated the co-expression of CD3 and CD56 surface antigens on fractionated $LAK1^+$ and $LAK1^-$ cultured TIL. As shown in Figure 1, $CD3^+$ $CD56^+$ cells represented about 35% of $LAK1^+$ TIL (Figure 1a) and 17% of $LAK1^-$ cells (Figure 1b) respectively. Moreover, TIL cultured in 25 U $ml^{-1}$ rIL2 contained predominantly $CD4^+$ cells, whereas a higher percentage of $CD8^+$ lymphocytes was observed in the presence of 1,000 U $ml^{-1}$ rIL2 (Table I). When the lytic activity of unfractionated TIL was analysed, using autologous and allogeneic tumours, and the K562 cell line as targets, a specific cytotoxicity was observed only in two cases. No significant difference between low and high doses of the lymphokine in terms of induction of cytotoxicity against fresh autologous tumours was evident in most cases; conversely NK activity was constantly higher after culturing effector cells with 1,000 U $ml^{-1}$ rIL2 (not shown). We could not detect any relationship between phenotypic or functional pattern of TIL and their source (lung or kidney cancer).

### *Cloning of TIL*

To characterise further the effector cells among TIL, clonal analysis by limiting dilution was carried out, and the growing clones were analysed with regard to their lytic activity, lymphokine production and expression of LAK1 antigen. Ten clones (out of 21 tested) did not exert any type of cytotoxicity whereas eleven clones displayed high lytic potential in LDCC (Table II). The majority of these clones (9/11) were $LAK1^+$, six were also $CD4^+$ while four expressed the CD8 antigen and one was $CD4^-CD8^-$. Surprisingly, none of the 11 clones showing LDCC was able to kill the fresh allogeneic tumour nor the NK-resistant Mel10 and Epa1 cell lines. Conversely, two clones displayed a significant killing of the autologous tumour and both of them lacked the LAK1 antigen (Figure 2a). It is of note that only one clone, showing $CD3^+WT31^-$ phenotype, killed the NK-sensitive K562 cell line (Figure 2a and Table II). Moreover, we observed that all $LAK1^+$ TIL clones produced high amounts of both α-TNF and γ-IFN upon stimulation with PHA, whereas the $LAK1^-$ did not (Figure 2b).

**Figure 1** Two-colour cytofluorometric analysis of CD3 (green fluorescence, *x* axis) and CD56 (red fluorescence, *y* axis) antigen expression on fractionated $LAK1^+$ **a**, and $LAK1^-$ **b**, TIL. Cells were fractionated by immunorosetting and stained with Fluorescein-conjugated anti-CD3 (Leu4) followed by Phycoerythrin-conjugated anti-CD56 (Leu19) MoAbs and run on a FACStar Plus.

### *Fractionation of $LAK1^+$ and $LAK1^-$ TIL*

In order to confirm the hypothesis that LAK1 antigen might dissect TIL into specific CTL and NK/LAK cells, TIL from a RCC were fractionated into $LAK1^+$ and $LAK1^-$ cells by immunorosetting and tested for their lytic activity against autologous or allogeneic tumour targets and the K562 cell line. Lysis of the autologous tumour was mainly evident when $LAK1^-$ TIL, cultured both with low and high doses of rIL2, were used as effector cells. On the other hand LAK activity was present only when 1,000 U $ml^{-1}$ rIL2 were used, and was confined to the $LAK1^+$ subset (Table III). As shown

**Table I** Surface phenotype of TIL obtained from six lung and six kidney tumours and expanded in presence of rIL2

| *Days of culture*[a] | *CD4*$^+$ | *CD8*$^+$ | *LAK1*$^+$ | *CD56*$^+$ | *CD56*$^+$ *LAK1*$^+$ | *CD3*$^+$ *LAK1*$^+$ | *CD56*$^+$ *CD3*$^+$ |
|---|---|---|---|---|---|---|---|
| Day 0 | 45±6[b] | 27±7 | 28±7 | 12±6 | 4±2 | 20±7 | 2±1 |
| Day 7 = QIL2 25 U ml$^{-1}$ | 51±8 | 29±6 | 28±5 | 17±4 | 6±2 | 22±6 | 4±1 |
| Day 7 = QIL2 1,000 U ml$^{-1}$ | 40±7 | 39±8 | 36±6 | 23±4 | 9±4 | 29±6 | 9±1 |
| Day 14 + rIL2 25 U ml$^{-1}$ | 52±4 | 34±5 | 31±7 | 15±6 | 6±2 | 27±5 | 11±5 |
| Day 14 + rIL2 1,000 U ml$^{-1}$ | 30±6 | 54±7 | 46±5 | 31±6 | 14±3 | 34±7 | 19±6 |

[a]Cells were cultured up to 14 days in rIL2 at different concentrations. [b]Percentage positive cells. Mean values ± s.d.

**Table II** Phenotypic and functional analysis of cloned TIL from a renal cell carcinoma

| *Surface phenotype* | | | | | | *Cytolytic activity* | | | |
|---|---|---|---|---|---|---|---|---|---|
| *CD4*$^+$ | *CD8*$^+$ | *WT31*$^-$ | *LAK1*$^+$ | *CD45R*$^+$ | *CDW29*$^+$ | *LDCC* | *NK* | *LAK* | *CTL* |
| 14/21[a] | 6/21 | 1/21 | 11/21 | 1/21 | 20/21 | 11/21[b] | 1/21 | 0/21 | 2/21 |

[a]Number of positive clones. [b]Number of clones showing lytic activity (> 20% of specific lysis at the E:T ratio of 10:1).

**Figure 2** Natural killer (NK) *vs* specific lysis (CTL) (**a**, % specific lysis) and production of gamma-interferon (γ-IFN) (**b**, IU ml$^{-1}$) *vs* tumour necrosis factor alpha (α-TNF) (**b**, pg ml$^{-1}$) of the 11 clones showing LDCC compared with their LAK1 phenotype (LAK1$^+$, □; LAK1$^-$, ■).

in Figure 1b, LAK1$^-$ subset was mainly represented by CD3$^+$CD56$^-$ cells, whereas one-third of LAK1$^+$ TIL (Figure 1a) was CD3$^+$CD56$^+$. These data further suggest that LAK1$^+$ TIL are enriched in non-MHC-restricted cytotoxic T lymphocytes bearing NK cell markers (CD56). No significant difference in terms of NK activity was observed between LAK1$^-$ and LAK1$^+$ cells. Similar results were obtained using TIL from a lung carcinoma (not shown).

**Table III** Cytotoxic activity of fractionated LAK1$^+$ and LAK1$^-$ TIL

| | *LU per 10$^6$ cells*[a] | |
|---|---|---|
| | *Autologous* | *Allogeneic* |
| 25 U ml$^{-1}$ | | |
| LAK1$^+$ | 2.1 | 5.8 |
| LAK1$^-$ | 20.5 | 2.2 |
| 1,000 U ml$^{-1}$ | | |
| LAK1$^+$ | 5.2 | 12.4 |
| LAK1$^-$ | 66.8 | <1 |

[a]One LU was defined as the number of effector cells needed to lyse 20% of 5 × 10$^3$ targets. Data are referred to TIL from a RCC grown in the presence of 25 U ml$^{-1}$ or 1,000 U ml$^{-1}$ rIL2.

### Discussion

The aim of our study was to determine whether LAK1 antigen is able to distinguish among TIL obtained from different solid tumours, two populations of effector cells with either specific or LAK activity. We first analysed the distribution of the LAK1 molecule on TIL, and found that about 25% of freshly derived TIL were weakly stained with anti-LAK1 MoAb. As previously reported (Zocchi *et al.*, 1989), the LAK1 antigen showed a bimodal distribution among normal PBL, defining two subsets of cells with different reactivity with the MoAb (in terms of fluorescence intensity). Most brightly stained LAK1$^+$ lymphocytes co-expressed the CD2 and CD56 antigens but lacked CD3, whereas a fraction (50%) of weakly stained LAK1$^+$ cells co-expressed also CD3. Since the vast majority of TIL belonged to the T cell lineage, the finding of a weak reactivity with anti-LAK1 MoAb was consistent with the above mentioned data. After culture in rIL2 the percentage of LAK1$^+$ TIL increased up to 50% and these cells co-expressed either CD3 (50–60%) or CD56

(20–30%) antigens (Table I). In addition, among LAK1$^+$ cells, about one-third co-expressed the CD3 and CD56 molecules. As the expression of LAK1 antigen is not induced by rIL2, the increase of LAK1$^+$ cells suggests that under these culture conditions a population of LAK cells was expanding. In agreement with this observation, the LAK activity of TIL cultures, when evaluated at different time intervals, increased over the following two weeks (not shown). Moreover, TIL cultured in 25 U ml$^{-1}$ rIL2 contained predominantly CD4$^+$ cells, whereas a higher percentage of CD8$^+$ lymphocytes was observed in the presence of 1,000 U ml$^{-1}$ rIL2. The lytic pattern of cultured TIL was quite variable and in only two cases (out of 12) was a specific cytotoxicity observed. We could not detect any significant difference between low and high doses of rIL2 in terms of killing of fresh tumour targets, while NK activity seemed to be dose-related. In these experiments we used two concentrations of the lymphokine in order to rule out the possibility that high doses could mask any specific lytic activity. No relationship between phenotypic or functional pattern of TIL and their source (lung or kidney cancer) was detected.

A clonal analysis of TIL from a RCC showed that the majority of the clones were CD4$^+$, LAK1$^+$ and displayed high lytic potential in LDCC (Table II). Nevertheless only one clone was able to kill K562 target cells and none of the clones tested killed the allogeneic tumour. Interestingly, the clone with NK activity showed a CD3$^+$WT31$^-$ phenotype consistent with data from other laboratories supporting the notion that gamma$^+$ delta$^+$ T lymphocytes are able to exert non MHC-restricted cytotoxicity (Borst *et al.*, 1987). Two clones displayed a slight but significant specific lysis and both of them were LAK1$^-$. Furthermore, unlike LAK1$^-$ clones, all LAK1$^+$ TIL clones produced high amounts of α-TNF and γ-IFN upon stimulation with PHA (Figure 2b). Recently, it has been reported that tumour cell lines stimulate LAK cells to produce factors, in particular γ-IFN and TNF, with slow acting cytotoxic activity (Chong *et al.*, 1989).

LAK1$^+$ clones might exert their anti-tumour activity throughout this mechanism rather than killing tumour targets by direct cell to cell contact. It is of note that LAK1$^-$ clones did not produce either lymphokine, suggesting that α-TNF and γ-IFN are not responsible for the autologous tumour cell lysis displayed by these clones. The different capability of producing α-TNF and γ-IFN showed by LAK1$^+$ and LAK1$^-$ cloned TIL further support the concept that LAK1 antigen could divide TIL into two populations with different functional properties.

On the basis of these data we were able to hypothesise that, among TIL, specific cytotoxic lymphocytes were confined to the LAK1$^-$ population, whereas cells expressing LAK1 antigen were responsible for LAK activity. In order to substantiate this hypothesis, TIL from a RCC were fractionated into LAK1$^+$ and LAK1$^-$ cells and tested for their lytic activity against autologous or allogeneic tumour targets and the NK-sensitive K562 cell line. Lysis of the autologous tumour was mainly evident when LAK1$^-$ TIL, cultured with both low and high doses of rIL2, were used as effector cells. On the other hand LAK activity was present only when 1,000 U ml$^{-1}$ rIL2 were used and was confined to the LAK1$^+$ subset (Table III). No significant difference in terms of NK activity was observed among the LAK1$^+$ and LAK1$^-$ subsets (not shown).

On the basis of our results, we conclude that TIL can be dissected by LAK1 MoAb into two subpopulations with either specific or LAK activity. It is not clear what role these cells play in the control of tumour growth and metastasis. Nevertheless, the LAK1 MoAb may represent a useful tool for studying the relationship between T lymphocytes and tumour cells. Further studies to define cellular and biological characteristics of TIL, both at the population and at the clonal level, are in progress.

We thank Dr Silvia Heltai for FACS analysis, and Dr M. Maffezzini and Dr G. Di Credico (Surgical Staff, Ospedale San Raffaele) for providing tumour specimens.

### References

BELLDEGRUN, A., MUUL, L.M. & ROSENBERG, S.A. (1988). Interleukin-2 expanded tumor-infiltrating lymphocytes in human renal cell cancer: isolation, characterization and antitumor activity. *Cancer Res.*, **48**, 206.

BORST, J., VAN DE GRIEND, R.J., VAN OOSTOVEEN, J.W. & 5 others (1987). A T-cell receptor gamma/CD3 complex found on cloned functional lymphocytes. *Nature*, **325**, 683.

CHONG, A.S.F., SCUDERI, P., GRIMES, W.J. & HERSH, E.M. (1989). Tumor targets stimulate IL-2 activated killer cells to produce interferon and tumor necrosis factor. *J. Immunol.*, **142**, 2133.

COLOTTA, F., BERSANI, L., LAZZARIN, A., POLI, G. & MANTOVANI, A. (1985). Rapid killing of Actinomycin-D-treated tumor cells by human monocytes. II. Cytotoxicity is independent of secretion of reactive oxigen intermediates and is suppressed by protease inhibitors. *J. Immunol.*, **134**, 3524.

HENNEY, C. (1971). Quantitation of the cell-mediated immune response. I. The number of cytolytically active mouse lymphoid cells induced by immunization with allogeneic mastocytoma cells. *J. Immunol.*, **107**, 1558.

ITOH, K., PLATSOUCAS, C.D. & BALCH, C. (1988). Autologous tumor specific cytotoxic T lymphocytes in the infiltrate of human metastatic melanomas. *J. Exp. Med.*, **168**, 1419.

LANIER, L.L. & PHILLIPS, J.H. (1986). Evidence for three types of human cytotoxic lymphocytes. *Immunol. Today*, **7**, 132.

MELIOLI, G., MERLI, A., FERRINI, S., MINGARI, M.C. & MORETTA, L. (1985). Phenotype and functional heterogeneity of human T cell clones producing gamma-interferon. *La Ricerca Clin. Lab.*, **15**, 47.

MORETTA, A., PANTALEO, G., MORETTA, L., MINGARI, M.C. & CEROTTINI, J.C. (1983). Quantitative assessment of the pool size and subset distribution of cytolytic T lymphocytes within human resting or alloactivated peripheral blood T cell populations. *J. Exp. Med.*, **158**, 571.

MUUL, L.M., SPIESS, P.S., DIRECTOR, E.P. & ROSENBERG, S.A. (1987). Identification of specific cytolytic immune responses against autologous tumor in humans bearing malignant melanoma. *J. Immunol.*, **138**, 989.

RAMARLI, D., FOX, D.A. & REINHERZ, E.L. (1987). Selective inhibition of interleukin-2 gene function following thymocyte antigen/major histocompatibility complex receptor crosslinking: possible thymic selection mechanism. *Proc. Natl Acad. Sci. USA*, **84**, 8598.

TASWELL, C. (1981). Limiting dilution assays for the determinations of immunocompetent cell frequencies. *J. Immunol.*, **126**, 1614.

WHITESIDE, T.L., HEO, D.S. & HERBERMAN, R.B. (1988). Characterization of novel anti-tumor effector cells in long-term cultures of human tumor infiltrating lymphocytes. *Transpl. Proc.*, **2**, 347.

ZOCCHI, M.R., BOTTINO, C., FERRINI, S., MORETTA, L. & MORETTA, A. (1987). A novel 120 kD surface antigen expressed by a subset of human lymphocytes. Evidence that lymphokine-activated killer cells express this molecule and use it in their effector function. *J. Exp. Med.*, **166**, 319.

ZOCCHI, M.R., POGGI, A., GIANAZZA, E., MARIANI, S. & RUGARLI, C. (1989). Identification of a new surface molecule expressed by human LGL and LAK cells: production of a specific monoclonal antibody and comparison with other NK/LAK markers. *Cell. Immunol.*, **124**, 144.